# Targeting Adenosine Receptors for the Treatment of Cardiac Fibrosis

**DOI:** 10.3389/fphar.2017.00243

**Published:** 2017-05-05

**Authors:** Elizabeth A. Vecchio, Paul J. White, Lauren T. May

**Affiliations:** ^1^Monash Institute of Pharmaceutical Sciences, Monash University, ParkvilleVIC, Australia; ^2^Department of Pharmacology, Monash University, ParkvilleVIC, Australia

**Keywords:** adenosine, adenosine A_2B_ receptor, cardiac fibrosis, fibroblast, collagen synthesis, cAMP, myocardial infarction, heart failure

## Abstract

Adenosine is a ubiquitous molecule with key regulatory and cytoprotective mechanisms at times of metabolic imbalance in the body. Among a plethora of physiological actions, adenosine has an important role in attenuating ischaemia-reperfusion injury and modulating the ensuing fibrosis and tissue remodeling following myocardial damage. Adenosine exerts these actions through interaction with four adenosine G protein-coupled receptors expressed in the heart. The adenosine A_2B_ receptor (A_2B_AR) is the most abundant adenosine receptor (AR) in cardiac fibroblasts and is largely responsible for the influence of adenosine on cardiac fibrosis. *In vitro* and *in vivo* studies demonstrate that acute A_2B_AR stimulation can decrease fibrosis through the inhibition of fibroblast proliferation and reduction in collagen synthesis. However, in contrast, there is also evidence that chronic A_2B_AR antagonism reduces tissue fibrosis. This review explores the opposing pro- and anti-fibrotic activity attributed to the activation of cardiac ARs and investigates the therapeutic potential of targeting ARs for the treatment of cardiac fibrosis.

## Introduction

Cardiac fibroblasts form the largest population of interstitial cells in the adult mammalian heart (Chen and Frangogiannis, 2013). They have an essential role in the regulation of the extracellular matrix (ECM), which is crucial for maintaining the structural integrity of the myocardium and for electro-mechanical signal transduction (Camelliti et al., 2004; Souders et al., 2009). Cardiac fibroblasts are regulated by various mechanical and hormonal stimuli, in particular growth factors such as angiotensin II (ANGII) and the cytokine transforming growth factor β (TGFβ). ANGII and TGFβ can activate fibroblast cell-surface receptors to promote differentiation to myofibroblasts, the pro-fibrogenic phenotype that express the contractile protein α-smooth muscle actin (α-SMA) and exhibit enhanced secretory, migratory and proliferative properties (Schnee and Hsueh, 2000; Petrov et al., 2002; Leask, 2007; Porter and Turner, 2009; Lu and Insel, 2014). Following a myocardial infarction (MI), fibroblasts promote essential matrix deposition for proper tissue repair and scar formation to ensure structural integrity of the infarct zone. However, aberrant ECM deposition and excessive myofibroblast accumulation extending beyond the area of the original insult is responsible for maladaptive fibrosis leading to cardiac dysfunction, a hallmark feature of heart failure pathophysiology (See et al., 2005; Segura et al., 2012; Ferrari et al., 2016). Heart failure remains a major cause of mortality and morbidity in the western world with an estimated 50% 5 years survival rate after diagnosis (Mozaffarian et al., 2016). This highlights both the limitations of current therapeutic management and the crucial need for new and innovative therapies for the treatment and prevention of heart failure. Extracellular nucleotides and nucleosides have recently been implicated as important mediators of fibroblast homeostasis and as such purinergic signaling has been investigated for its role in cardiac fibrosis. AMP catabolites, including inosine and oxypurines have also been shown to contribute to cardiac fibrosis and diastolic stiffening in some animal models of heart failure (Paolocci et al., 2006). The role of nucleotide (ATP, ADP, UTP) signaling in tissue fibrosis has been comprehensively reviewed previously (Lu and Insel, 2014; Ferrari et al., 2016; Novitskaya et al., 2016), therefore the current review will focus the modulation of cardiac fibrosis mediated by the nucleoside adenosine and adenosine receptors (ARs).

## Adenosine Signaling in the Heart

**FIGURE 1:**
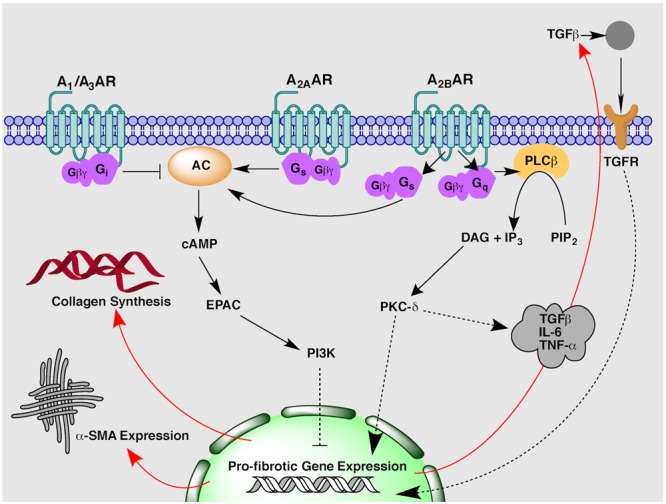
**An overview of proposed adenosine receptor-mediated intracellular signaling pathways implicated in the regulation of cardiac fibrosis**.

Adenosine is a ubiquitous purine nucleoside that is an important regulator of cardiac function. Adenosine is described as a ‘retaliatory metabolite’ owing to its enhanced local release and ability to restore energy balance during times of cellular and metabolic stress (Newby, 1984; Shyrock and Belardinelli, 1997). The well-characterized cytoprotective actions have resulted in large clinical trials for adenosine and adenosine derivatives for the treatment of ischaemia-reperfusion injury post-MI (Kopecky et al., 2003; Ross et al., 2005; Forman et al., 2006). In addition to a clear role in cardioprotection, adenosine exerts a multitude of actions on the physiological regulation of the heart, including coronary vasodilation, heart rate control and AV nodal conduction, angiogenesis, myocardial hypertrophy and remodeling and fibrosis (Auchampach and Bolli, 1999; Peart and Headrick, 2007; Headrick et al., 2013). The myriad of cardiovascular effects stimulated by adenosine occur via activation of specific cell surface ARs. The AR family is comprised of four Class A G protein-coupled receptors (GPCRs), the A_1_, A_2A_, A_2B_ and A_3_ARs. They exert distinct pharmacological actions through differential coupling to intracellular G proteins; the A_1_AR and A_3_AR preferentially activate G_i/o_ proteins to inhibit adenylyl cyclase activity and subsequent cAMP production, while the A_2A_AR and A_2B_AR preferentially stimulate G_s_ proteins to activate adenylyl cyclase activity and increase cAMP accumulation (**Figure 1**) (Fredholm et al., 2001). The A_2B_AR has also been shown to stimulate robust G_q/11_ protein activation in some cell types (Feoktistov and Biaggioni, 1997; Linden et al., 1999). ARs, and the A_2B_AR in particular, have also been shown to couple to additional transmembrane and intracellular proteins, which may influence downstream signal transduction (Mundell and Benovic, 2000; Fredholm et al., 2001; Sun and Huang, 2016). All four ARs are expressed in the heart and synchronous activation of multiple subtypes results in both complementary and opposing signal transduction for the fine-tuned regulation of cardiac function. Interestingly, both pro- and anti-fibrotic actions have been attributed to AR activation, which highlights both the complexity and ensuing challenges faced when targeting ARs for the treatment of cardiac fibrosis (Chan and Cronstein, 2009; Cronstein, 2011; Karmouty-Quintana et al., 2013). To date, the preponderance of evidence has implicated the A_2B_AR in cardiac fibrosis (Epperson et al., 2009; Headrick et al., 2013; Novitskaya et al., 2016). Therefore, this review will explore the current understanding of the role of AR signaling in augmenting or attenuating cardiac fibrosis, with a focus on the predominant subtype implicated, the A_2B_AR.

## A_2B_AR-Mediated Anti-Fibrotic Signal Transduction

Studies in isolated rat cardiac fibroblasts first proposed the A_2B_AR as the subtype responsible for mediating adenosine’s inhibitory actions on fetal calf serum-stimulated fibroblast proliferation (Dubey et al., 1997) and collagen and protein synthesis (Dubey et al., 1998). The role of the A_2B_AR in adenosine-mediated anti-fibrotic signal transduction was later confirmed via antisense oligonucleotide A_2B_AR silencing, which resulted in increased cell proliferation and basal collagen synthesis in cardiac fibroblasts (Dubey et al., 2001b). Similarly, A_2B_AR overexpression had the opposite effect, significantly decreasing collagen and protein synthesis (Chen et al., 2004). The second messenger cAMP, has been shown to have a central role in inhibiting fibroblast and myofibroblast activity (Swaney et al., 2005; Lu et al., 2013). Accordingly, A_2B_AR-mediated cAMP accumulation stimulated in fibroblasts by the non-selective AR agonist 5′-*N-*ethylcarboxamidoadenosine (NECA) (Epperson et al., 2009) can reduce ANGII-stimulated collagen synthesis via an exchange factor directly activated by cAMP (Epac) and phosphoinositol-3 kinase (PI3K) dependent pathway (**Figure 1**) (Villarreal et al., 2009). In addition to effects on collagen synthesis, A_2B_AR stimulation has been shown to decrease mRNA expression of pro-fibrotic gene markers including collagen I and connective tissue growth factor (CTGF) (Vecchio et al., 2016). Of specific importance to ARs, a positive feedback loop has been identified whereby β-adrenoceptor-stimulated cAMP can be secreted by fibroblasts or cardiac myocytes and metabolized in the extracellular space to adenosine to activate A_2_ARs, thus exerting further inhibitory effects on fibroblast growth and function (Dubey et al., 2001a; Sassi et al., 2014).

Commensurate with the *in vitro* findings, an *in vivo* study in rats demonstrated chronic administration of the stable adenosine analog, 2-chloroadenosine (CADO) or the adenosine uptake inhibitor, dipyridamole, initiated 1 week after permanent ligation of the left anterior descending (LAD) coronary artery, protected against cardiac remodeling and reduced markers of fibrosis such as collagen volume fraction and matrix metalloproteinase gene expression (Wakeno et al., 2006). The effects of CADO on fibrotic and haemodynamic parameters were abolished in the presence of the selective A_2B_AR antagonist MRS1754, but not selective antagonists for the other AR subtypes (Wakeno et al., 2006). Together, these studies suggest a salutary effect of A_2B_AR activation on cardiac fibrosis, an effect which may be lost upon A_2B_AR downregulation as observed in hearts taken from human patients with chronic heart failure (Asakura et al., 2007).

## A_2B_AR-Mediated Pro-Fibrotic Signal Transduction

While the majority of *in vitro* studies have identified an anti-fibrotic role for the A_2B_AR, recent studies have demonstrated A_2B_AR blockade appears to be beneficial within *in vivo* models of cardiac remodeling and fibrosis. In an *in vivo* mouse model of MI involving permanent coronary artery ligation, chronic administration of a novel, highly selective A_2B_AR antagonist, GS-6201, significantly reduced cardiac enlargement and dysfunction compared to vehicle-treated mice (Toldo et al., 2012). Similarly in an *in vivo* rat myocardial ischaemia-reperfusion model, GS-6201 improved ejection fraction and decreased fibrosis in the non-infarct and border zones with the greatest effect observed when GS-6201 was given 1 week rather 1 day after MI (Zhang et al., 2014). A pro-fibrotic role for the A_2B_AR has been supported by a study in A_2B_AR knock-out (A_2B_AR^-/-^) mice that demonstrate the A_2B_AR contributes to post-infarction heart failure (Maas et al., 2008). A_2B_AR^-/-^ mice had improved end diastolic pressure and reduced interstitial fibrosis when compared to wild-type mice 8 weeks after permanent left coronary ligation. Systolic blood pressure and infarct size remained the same between knock-out and wild-type animals suggesting the A_2B_AR contributes to heart failure pathology via post-infarction remodeling and reactive fibrosis rather than acute cardioprotection (Maas et al., 2008). The mechanism underlying the pro-fibrotic activity of the A_2B_AR may involve the pro-inflammatory effects mediated by this AR subtype. Blockade of the A_2B_AR inhibits caspase-1 activity and leukocyte infiltrate (Toldo et al., 2012), and attenuates secretion of pro-fibrotic and pro-inflammatory mediators such as TGFβ, tumor necrosis factor α (TNF-α) and interleukin-6 (IL-6) post-MI via a PKC-δ pathway (**Figure 1**) (Feng et al., 2009; Toldo et al., 2012; Zhang et al., 2014). A pro-inflammatory role of the A_2B_AR is reported by studies in other organ systems, in particular the lung where elevated adenosine concentrations and A_2B_AR activity promotes chronic fibrosis and inflammation in asthma and chronic obstructive pulmonary disease (Sun, 2006; Chan and Cronstein, 2009; Zhou et al., 2009; Karmouty-Quintana et al., 2013). Given the inflammatory response is intricately linked to the regulation of tissue fibrosis, it is perhaps unsurprising therefore, that the A_2B_AR has been implicated as a promoter of cardiac fibrosis *in vivo* (Ham and Rees, 2008; Kong et al., 2013; Stuart et al., 2016).

## A_1_AR Modulation of Cardiac Fibrosis

The protective role of A_1_AR activation in cardiac remodeling appears to be largely attributed to the beneficial effects on cardiomyocyte hypertrophy rather than effects on fibrosis (Liao et al., 2003; Sassi et al., 2014; Chuo et al., 2016). A study using a non-selective adenosine analog (CADO) in mice subject to 4 weeks of chronic pressure overload via transverse aortic constriction (TAC), demonstrated reduced myocardial and perivascular fibrosis and hypertrophy compared to saline-treated mice (Liao et al., 2003). Attenuation of myocardial hypertrophy was A_1_AR-mediated, as the anti-hypertrophic effects were reversed in the presence of an A_1_AR-selective antagonist. As similar antagonist studies were not reported for measures of cardiac fibrosis (Liao et al., 2003), it cannot be ruled out that the anti-fibrotic effects were mediated by another AR subtype, in particular the A_2B_AR. However, recent studies using more A_1_AR-selective agonists do suggest an involvement of the A_1_AR in cardiac fibrosis. A study of heart failure in dogs demonstrated capadenoson, an A_1_AR partial agonist, decreased interstitial fibrosis (Sabbah et al., 2013). Similarly, activation of the A_1_AR with a selective agonist N^6^-cyclopentyladenosine (CPA), attenuated left ventricular collagen content and markers of fibrosis in response to α_1_-adrenergic stimulation *in vivo* (Puhl et al., 2016).

Activation of the A_1_AR has been recognized as central to the acute cardioprotective actions of adenosine (McIntosh and Lasley, 2012; Headrick et al., 2013). In agreement, overexpression of the A_1_AR protects mice against acute ischaemic events, with cardiac infarct size markedly reduced in transgenic compared to wild-type animals (Yang et al., 2002). Paradoxically, however, chronic A_1_AR cardiac overexpression in older mice (20 weeks) has been associated with enhanced baseline cardiac fibrosis and dilated cardiomyopathy (Funakoshi et al., 2006). Additionally, a study investigating myocardial fibrosis secondary to chronic renal failure demonstrated that an A_1_AR-selective antagonist, SLV320, normalized cardiac collagen I and III content in the hearts of rats that had undergone a nephrectomy (Kalk et al., 2007). These studies may suggest chronic A_1_AR stimulation reduces the cardiac resistance to non-ischaemic stress and may promote fibrosis, however, the conflicting evidence highlights the need for further studies to fully elucidate the role of this AR subtype in cardiac fibrosis.

## A_2A_AR Modulation of Cardiac Fibrosis

Separating the contribution of A_2B_AR-mediated fibrotic signaling from that of A_2A_AR activation has been difficult owing to the paucity of early subtype selective agonists and antagonists. Genetic alteration of the A_2A_AR demonstrated that cardiac-specific overexpression of the A_2A_AR in mice was protective against pressure-induced heart failure, attenuating fibrosis and improving cardiac function (Hamad et al., 2012). A more recent study demonstrated high A_2A_AR expression in mouse cardiac fibroblasts stimulated the accumulation of the anti-fibrotic second messenger cAMP (Sassi et al., 2014), though perhaps to a lesser extent than the A_2B_AR (Epperson et al., 2009). Combined with the known anti-inflammatory actions of the A_2A_AR in the heart (Linden, 2001; Haskó et al., 2008), there is certainly valid grounds to suggest that A_2A_AR signaling would attenuate cardiac fibrosis. However, further work is needed to clarify the exact role of A_2A_AR, as stimulation of this receptor subtype has also been demonstrated to have pro-fibrotic effects in other organs such as the liver and skin (Chan et al., 2006a,b; Perez-Aso et al., 2014).

## A_3_AR Modulation of Cardiac Fibrosis

Comparatively few studies have investigated the role of the A_3_AR in cardiac fibrosis, which is unsurprising given early studies examining the A_3_AR (and A_1_AR) expressed on isolated rat cardiac fibroblasts suggested these receptors to be of lesser functional importance than the A_2_ARs (Chen et al., 2004). The A_3_AR was investigated for its involvement in protecting against maladaptive cardiac hypertrophy and fibrosis on the basis that ecto-5′-nucleotidase (CD73; catalyzes the conversion of extracellular AMP to adenosine) deficiency exacerbated myocardial hypertrophy and heart failure in TAC mice (Xu et al., 2008). Contrary to hypothesis, A_3_AR knock-out mice actually had reduced left ventricular hypertrophy, fibrosis and dysfunction after 5 weeks of TAC compared to wild-type animals. There was no effect of A_3_AR deletion on parameters in the unstressed heart, suggesting the A_3_AR has a deleterious role in cardiac fibrosis only in response to chronic pressure overload (Lu et al., 2008). In agreement, a recent study using a uninephrectomy and high salt-induced model of hypertension in mice, demonstrated that genetic abrogation of the A_3_AR resulted in significantly less cardiac hypertrophy and fibrosis compared to wild-type animals (Yang et al., 2016). These studies suggest A_3_AR antagonism may be a valid therapeutic approach to prevent chronic pressure overload-hypertrophy and fibrosis, however, further studies are warranted.

## Conclusion and Future Directions

Cardiac fibrosis is an important determinant of left ventricular dysfunction and remodeling following MI and is a hallmark of heart failure pathology, which is associated with an extremely high rate of mortality (See et al., 2005; Segura et al., 2012). It is therefore crucial to find new therapeutic approaches to prevent and ideally reverse underlying cardiac fibrosis in order to modify the disease progression of heart failure. Purinergic signaling downstream of AR activation represents one such novel strategy to influence fibrosis homeostasis, however, much work is still needed to clarify the exact role of the receptor subtypes involved. A central question that remains is how the same receptor subtype can have both pro- and anti-fibrotic activity. The opposing effects as outlined in this review, may reflect differences in underlying disease pathology due to the type and duration of cardiac insult; whereby AR activation appears to be largely anti-fibrotic in acute ischaemic events but potentially pro-fibrotic under conditions of chronic myocardial stress. This supposition is supported by studies of adenosine’s involvement in fibrosis of other organ systems (Karmouty-Quintana et al., 2013). In the lung, A_2B_AR stimulation is protective in acute-bleomycin-induced lung injury but actually promotes fibrosis in chronic models of lung disease (Zhou et al., 2009, 2011). Similarly in the kidney, A_2B_AR activation is beneficial in attenuating acute kidney injury (Grenz et al., 2012) but prolonged A_2B_AR signaling increases interstitial fibrosis and collagen deposition in renal tissue (Roberts et al., 2014a,b). The exact mechanism behind these paradoxical effects requires further elucidation, but may reflect changes in differential receptor coupling with changes in cellular background as the disease progresses. Certainly, this idea is readily foreseeable for the A_2B_AR with its high degree of plasticity and ability to couple to multiple G proteins and intracellular signaling cascades (**Figure 1**) (Cohen et al., 2010). In addition, it should be noted a great deal of our understanding of adenosine’s role in cardiac fibrosis, in particular downstream of A_2B_AR, has come from *in vitro* studies. This may not reflect the true course of disease progression *in vivo* due to the exclusion of the inflammatory response and loss of organ complexity including cross-talk with other cell types. Therefore, while AR signaling appears to be a promising target in cardiac fibrosis, further studies are needed to fully appreciate the potential of AR therapeutics in heart failure and underlying fibrosis.

## Author Contributions

EV drafted the manuscript. PW and LM made substantial contribution to the writing. EV, PW, and LM provided critical revision of the manuscript and approved it for publication.

## Conflict of Interest Statement

The authors declare that the research was conducted in the absence of any commercial or financial relationships that could be construed as a potential conflict of interest.
